# VMAT-Based Planning Allows Sparing of a Spatial Dose Pattern Associated with Radiation Pneumonitis in Patients Treated with Radiotherapy for a Locally Advanced Lung Cancer

**DOI:** 10.3390/cancers14153702

**Published:** 2022-07-29

**Authors:** Vincent Bourbonne, Francois Lucia, Vincent Jaouen, Julien Bert, Olivier Pradier, Dimitris Visvikis, Ulrike Schick

**Affiliations:** 1 Department of Radiation Oncology, University Hospital, 29200 Brest, France; francois.lucia@chu-brest.fr (F.L.); olivier.pradier@chu-brest.fr (O.P.); ulrike.schick@chu-brest.fr (U.S.); 2 LaTIM UMR 1101 INSERM, University Brest, 29200 Brest, France; vjaouen@univ-brest.fr (V.J.); julien.bert@univ-brest.fr (J.B.); visvikis@univ-brest.fr (D.V.); 3 Institut Mines-Télécom Atlantique, 29200 Brest, France

**Keywords:** radiation pneumonitis, lung cancer, adaptive planning, cluster of voxels

## Abstract

**Simple Summary:**

A sub-region localized in the posterior right lung was significantly associated with the risk of grade ≥ 2 acute pulmonary toxicity in patients with locally advanced lung cancer treated with radiotherapy. Avoiding this sub-region with volumetric-arctherapy-based planification leads to a significant reduction of the predicted APT risk by reclassifying 43.2% (19/44) of the patients.

**Abstract:**

Introduction: In patients treated with radiotherapy for locally advanced lung cancer, respect for dose constraints to organs at risk (OAR) insufficiently protects patients from acute pulmonary toxicity (APT), such toxicities being associated with a potential impact on the treatment’s completion and the patient’s quality of life. Dosimetric planning does not take into account regional lung functionality. An APT prediction model combining usual dosimetry features with the mean dose (DMeanPmap) received by a voxel-based volume (Pmap) localized in the posterior right lung has been previously developed. A DMeanPmap of ≥30.3 Gy or a predicted APT probability (ProbAPT) of ≥8% were associated with a higher risk of APT. In the present study, the authors aim to demonstrate the possibility of decreasing the DMeanPmap via a volumetric arctherapy (VMAT)-based adapted planning and evaluate the impact on the risk of APT. Methods: Among the 207 patients included in the initial study, only patients who presented with APT of ≥grade 2 and with a probability of APT ≥ 8% based on the prediction model were included. Dosimetry planning was optimized with a new constraint (DMeanPmap < 30.3 Gy) added to the usual constraints. The initial and optimized treatment plans were compared using the t-test for the independent variables and the non-parametric Mann–Whitney U test otherwise, regarding both doses to the OARs and PTV (Planning Target Volume) coverage. Conformity and heterogeneity indexes were also compared. The risk of APT was recalculated using the new dosimetric features and the APT prediction model. Results: Dosimetric optimization was considered successful for 27 out of the 44 included patients (61.4%), meaning the dosimetric constraint on the Pmap region was achieved without compromising the PTV coverage (*p* = 0.61). The optimization significantly decreased the median DMeanPmap from 28.8 Gy (CI95% 24.2–33.4) to 22.1 Gy (CI95% 18.3–26.0). When recomputing the risk of APT using the new dosimetric features, the optimization significantly reduced the risk of APT (*p* < 0.0001) by reclassifying 43.2% (19/44) of the patients. Conclusion: Our approach appears to be both easily implementable on a daily basis and efficient at reducing the risk of APT. Regional radiosensitivity should be considered in usual lung dose constraints, opening the possibility of new treatment strategies, such as dose escalation or innovative treatment associations.

## 1. Introduction

(Chemo)-radiotherapy is the treatment of reference in locally advanced lung cancer (LALC) not amenable for surgery [1]. Modern radiation techniques, such as intensity-modulated radiation therapy (IMRT) and volumetric arctherapy (VMAT), allow a higher sparing of organs at risk (OAR) with lower doses without compromising the coverage of the planning tumour volume (PTV) [2,3]. Indeed, the VMAT dose sculpting proved to be very useful in the treatment of challenging tumor sites, for which the sparing of strictly close or even inner OARs was the goal in addition to achieving an excellent target coverage [4,5]. Despite the implementation of advanced RT techniques and of stricter dose–volume constraints, acute pulmonary toxicity (APT) remains frequent, with an approximate rate of 5–25% for grade ≥ 2 APT [6,7,8,9]. In a population treated with adjuvant immunotherapy, the APT rate rose to 33.9% for all grades and 3.3% for grades 3–4 APT [10,11].

The high frequency of APT could be explained by the functional heterogeneity of the lungs, these functional regions being mainly localized in the lower lungs [12,13,14,15]. On a daily basis, avoiding these regions could further reduce acute and late pulmonary toxicities [15]. However, although advanced RT techniques, such as VMAT, allow optimization, dosimetric planning does not currently take into account this regional lung heterogeneity. Indeed, the functional mapping of the lungs requires performing costly nuclear imaging, such as pulmonary ventilation and perfusion planar scintigraphy, or positron emission tomography/computed tomography (PET/CT) [15,16].

Based on a voxel-based analysis, as presented by Palma et al. [12,13], we previously identified and prospectively validated the possible role of a cluster of voxels localized in the posterior right lung. Patients with a high mean dose to this sub-region (DMean_Pmap_ ≥ 30.3Gy) were 4.7 times more likely to present with APT ≥ grade 2 when compared to the low-risk patients. Furthermore, when combining the Dmean_Pmap_ with ten other clinical and dosimetric features, a model was previously developed for the prediction of APT [17].

In patients with a predicted probability of APT ≥ grade 2 (Prob_APT_) ≥ 8%, the relative risk ratio rose to 12.6. Decreasing the Dmean_Pmap_ could, thus, lower the risk of APT. In this study, we aim to demonstrate the possibility of decreasing the Dmean_Pmap_ via VMAT-based adapted planning and evaluate its impact on the predicted risk of APT.

## 2. Materials and Methods

### 2.1. Population

Data from two separate cohorts were analyzed. The first cohort consisted of all patients treated with VMAT with curative intent for a histologically proven, locally advanced lung cancer (non-small cell or small-cell lung cancer) between 2015 and 2018. The second cohort consisted of the patients treated within the prospective trial TEFRARC (NCT03931356), evaluating the functional impact of lung radiotherapy. Characteristics of these 207 patients (165 from the retrospective and 42 from the prospective cohorts, respectively) were previously presented [9,17].

### 2.2. Radiation Sensitive Sub-Region

All computed tomographies (CTs) were registered to a thoracic phantom using a segmentation-based elastic registration via MIM Maestro (MIM v7.0.0, Cleveland, OH, USA). The segmentation used for registration was a hybrid volume of interest (VOI) consisting of the union of the lungs and the heart. The previously identified Pmap sub-region [17] was then transferred to the patient’s CT using the same elastic registration matrix but in reverse. The mean DICE coefficient between the phantom’s segmentation and each patient’s segmentation was evaluated. For the analysis, the doses were converted to biologically equivalent doses (BED) using the dose map conversion tool in MIM Maestro (MIM v7.0.0, Cleveland, OH, USA). Alpha/beta ratios of 3 for non-tumour volumes and 10 for tumour volumes were used. An explanatory flowchart is provided in Supplementary Figure S1.

### 2.3. Dosimetric Planning

Initial RT was delivered with a prescription dose of 60 to 66 Gy to the PTV, with 95% of the dose covering 95% of the prescription volume while respecting the usual dose constraints to the lungs, heart, and spine [18,19,20,21,22] (Supplementary Table S1).

Planning was optimized with the addition of specific constraints to the Pmap region, namely, a Dmean_Pmap_ < 30.3Gy (BED), while maintaining the PTV coverage and usual dose constraints. The initial Dmean_Pmap_ will now be defined as the Dmean_Pmap-Ini_, while the optimized Dmean_Pmap_ will be named Dmean_Pmap-Opti_.

Among the overall cohort, only patients that actually presented APT were considered (n = 45). Three cohorts were then defined, and differentiated on their APT risk, the Dmean_Pmap-Ini_, and the Dmean_Pmap-Opti_:

- Cohort 1: Patients who had a predicted risk of APT ≥ 8%, a Dmean_Pmap_ ≥ 30.3Gy, and in which dosimetric optimization was successful (Dmean_Pmap-Opti_ < 30.3Gy and respect to the PTV coverage and other dose constraints),
- Cohort 2: Patients who had a predicted risk of APT ≥ 8%, a Dmean_Pmap_ ≥ 30.3Gy, and in which dosimetric optimization was unsuccessful (Dmean_Pmap-Opti_ ≥ 30.3Gy and/or non-respect of the PTV coverage and other dose constraints).
- Cohort 3: Patients who had a predicted risk of APT ≥ 8% and a Dmean_Pmap_ < 30.3Gy. In Cohort 3, as the Dmean_Pmap_ was already inferior to 30.3Gy, the success of the optimization was defined by a ≥ 20% decrease of the Dmean_Pmap_ without compromising the PTV coverage.

The 8% threshold was based on a previous study [17], defining this cut-off as the most effective for the stratification of patients based on their APT risk. Dose constraints for the initial and optimized plannings are presented in Supplementary Table S1. The DICE score between the Pmap region and the PTV (DICE_PTV-Pmap_) was computed for each patient of Cohorts 1 and 2. The association between the DICE_PTV-Pmap_ and the success of optimization was analyzed using the Receiver Operative Characteristics (Area Under the Curve: AUC). An optimal cut-off was defined by maximizing the Youden index.

### 2.4. Dose Map Comparisons

Dose–volume histograms (DVHs) were calculated from the delivered RTPlan using the Pinnacle treatment planning system. Vx_y_ (Gy) will further be defined as the percentage of the volume of interest (VOI = y) receiving x dose (Gy). Dmean_y_ and Dmax_y_ correspond to the mean and maximal dose received by the VOI (y), respectively. On the homolateral (LungH) and contralateral (LungC) lungs, V5, V10, V13, V20, and V30, the Dmax and Dmean were collected. Regarding both lungs (2Lungs), VOI, V13, V20, V30, and the Dmean were considered. Finally, regarding the heart, V30, V40, and the Dmean were extracted. Dmean_Pmap_ and the PTV_95_ (percentage of the PTV receiving 95% of the prescription dose) were also analyzed. The significance tests used to estimate the inference between the two dose maps were the t-test for the independent variables and the non-parametric Mann–Whitney U test, otherwise.

The plan quality was assessed through the evaluation of conformity indices and heterogeneity indices. The conformity index (IC) is defined by $IC=\frac{PIV}{PTV}$, where the PIV (Prescription Isodose Volume) represents the volume receiving the prescription dose. The heterogeneity index (HIV) is defined by $HIV=\frac{D_{95\%}}{D_{5\%}}$, where D_x%_ represents the dose received by x% of the PTV.

### 2.5. Prediction Based on the Pmap Model

A Pmap model was previously developed combining 11 features, among which the Dmean_Pmap_ achieved the highest importance (35.8%) [17]. The remaining features were ranked by order of importance: Dmean_2Lungs_, V30_2Lungs_, Smoking Status, Mean Expiratory Volume/Second (MEVS), Chronic Obstructive Pulmonary Disease (COPD), V10_LungH_, AJCC Stage (American Joint Cancer Committee), V5_LungH_, Dmean_LungH_, andV40_Heart_. We applied the same Pmap model to the included patients using the optimized parameters. A new probability of APT was thus established. The pre-defined cut-off of 8% was used for patients’ classification.

## 3. Results

### 3.1. Population

**Figure 1:**
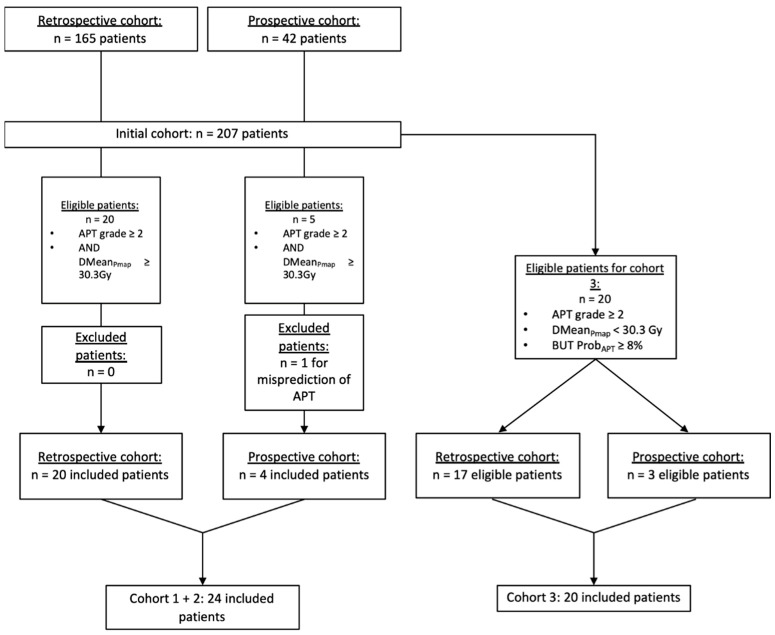
Flowchart of the patients’ selection. Abbreviations: APT: Acute Pulmonary Toxicity, DMean_Pmap_: mean dose received by the Pmap-region, Prob_APT_: the probability of an APT ≥ grade 2 based on the Pmap-prediction model.

Among the 207 initial patients, 25 and 20 patients were, respectively, considered eligible for Cohorts 1 + 2 and Cohort 3. Among the 25 patients from Cohorts 1 + 2 and before optimization, the mean Dmean_Pmap_ and median Dmean_Pmap_ were 40.4Gy (the standard deviation was 4.4Gy) and 39.3Gy (with a range of 30.6–60.2), respectively. The Pmap model correctly classified 96% of the patients (24/25), with one patient classified at low risk of APT (5.4%) despite further presenting with an APT ≥ grade 2 and a Dmean_Pmap_ of 34.7Gy. This patient was not considered for the rest of the analysis. Among the 20 patients eligible for Cohort 3, no patient was excluded, resulting in a mean Dmean_Pmap_ of 14.6Gy. A flowchart for the patients’ selection is presented in Figure 1.

### 3.2. Dosimetric Planning and Dose Map Comparisons

In Cohorts 1 and 2, the optimization was successful for 14 out of the 24 patients (58.3%) and Cohort 1 and Cohort 2 thus, respectively, consisted of 14 and 10 patients. Of the 24 included patients, the mean Dmean_Pmap_ significantly decreased from 39.4Gy (CI95% 36.4–41.9) to 30.1Gy (CI95% 27.8–34.9) while maintaining the PTV coverage. After the optimization, a non-significant increase of the PTV_95_ (*p* = 0.52) was observed at 95.4% (CI95% 92.7–96.4) vs. 94.8% (CI95% 93.5–96.6) before the optimization.

**Figure 2:**
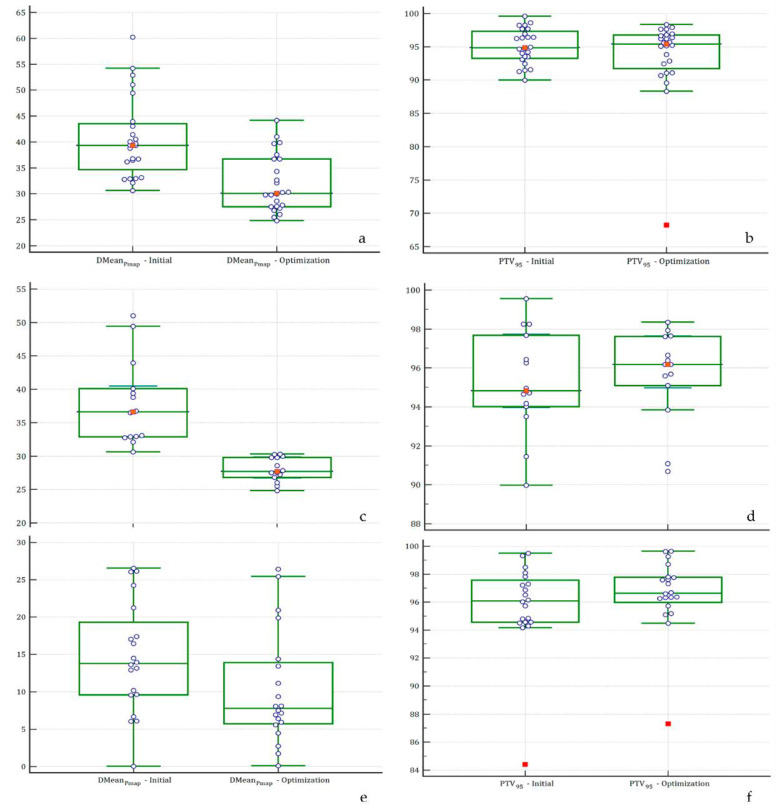
Changes in the DMean_Pmap_ and PTV coverage across each cohort; (*Y*-axis: percentage of coverage by the 95% isodose). DMean_Pmap_ (**a**) and PTV coverage (**b**) across Cohorts 1 + 2. The DMean_Pmap_ (**c**) and PTV coverage (**d**) across Cohort 1. The DMean_Pmap_ (**e**) and PTV coverage (**f**) across Cohort 3. Abbreviations: DMean_Pmap_: mean dose received by the Pmap region, PTV_95_: percentage of the Planning Target Volume (PTV) covered by 95% of the prescribed dose.

**Figure 3:**
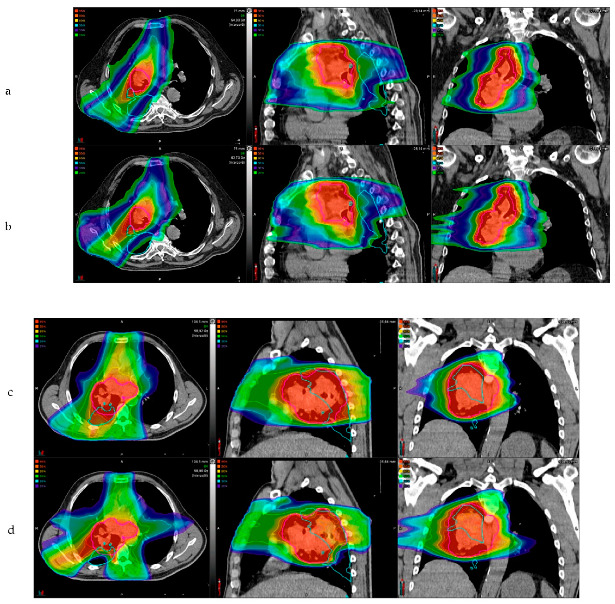
Examples of the initial and optimized dose maps. Abbreviations: COPD: Chronic Obstructive Disease, MEVS: Mean Expiratory Volume/Second, AJCC: American Joint Cancer Committee, PTV_95_: percentage of the planning target volume receiving 95% of the prescription dose, DMean_Pmap_: mean dose received by the Pmap volume, Prob_APT_: risk probability of acute pulmonary toxicity ≥ grade 2.

When focusing on Cohort 1, a significant decrease in the median Dmean_Pmap_ was also observed (*p* < 0.0001), from 36.6Gy (CI95% 32.9–40.5) to 27.7Gy (CI95% 26.7–29.8). The PTV coverage was conserved with no significant differences before (94.4%) and after (96.2%) the optimization (*p* = 0.65). Graphical comparisons between the initial planning and the optimized planning in each cohort are presented in Figure 2a,c (the Dmean_Pmap_) and Figure 2b,d (the PTV_95_ coverage). An example of the comparison between the two dose maps for both a successful patient and an unsuccessful patient is presented in Figure 3a–d, respectively.

No significant differences were observed for the main dose constraints to the lungs, heart, and spinal cord. For example, a non-significant increase for the V20_2Lungs_ and V30_2Lungs_ appeared for the optimized treatment planning, with the V20_2Lungs_ reaching 26.6% (vs. 22.2%, *p* = 0.22) and the V30_2Lungs_ reaching 18.4% (vs. 15.0%, *p* = 0.43) when compared to the initial dose maps. Regarding the Dmean_Heart_, a non-significant increase (*p* = 0.40) was also observed from 9.8Gy (CI95% 5.5–12.3) to 10.9Gy (CI95% 7.6–16.4). Similar results were observed for the sub-set of patients in which the optimization was successful. The detailed results for the dose constraints and PTV coverage are presented in Table 1. Regarding the evaluation of the treatment plans, no significant differences between the initial and the optimized plans were found for both the mean IC (0.96 +/− 0.03 vs. 0.94 +/− 0.06, *p* = 0.25) and the mean HIV (0.94 +/− 0.02 vs. 0.94 +/− 0.05, *p* = 0.77), respectively (Table 1).

Similar results were observed for the patients included in Cohort 3, with a reduction of the Dmean_Pmap_ from 14.6Gy to 10.3Gy (*p* = 0.08) without compromising the PTV coverage or other dosimetric features (Table 1, Figure 2e,f). Regarding the evaluation metrics, no significant differences were found (Table 1).

In Cohorts 1 and 2, with an AUC of 0.83 (*p* = 0.0001), the DICE_PTV-Pmap_ was significantly correlated with the success of the optimization (Supplementary Figure S2). The optimization was more likely to be successful in patients with a DICE_PTV-Pmap_ ≤ 0.15.

### 3.3. Model Prediction

The previously developed and validated Pmap model was applied to the 44 included patients with the new Dmean_Pmap-Opti_ and DVHs parameters. In Cohort 1, for the 14 patients in which the optimization was successful, 7/14 (50.0%) patients were reclassified at low risk of APT whereas initially predicted by the Pmap model at high risk. For the seven remaining patients (29.2%) from Cohort 1 and the 10 patients (41.6%) from Cohort 2 in which the optimization was unsuccessful, the prediction did not change with the exception of a single patient (Patient #3) from Cohort 2. The optimization, thus, significantly reduced the risk of APT (*p* = 0.0007) by reclassifying 33.3% (8/24) of the Cohort 1 + 2 patients.

Similarly, the risk of APT was significantly reduced in Cohort 3 by reclassifying 55.0% of the patients (*p* = 0.004), with 11 patients newly classified at low risk of grade ≥ 2 APT. The detailed results of the prediction model for each plan are presented in Table 2, while the individualized results are presented in Supplementary Table S2. A flowchart explaining the results is presented in Supplementary Figure S3.

**Table 1:** Dose–volume histograms’ parameters between the initial and optimized plannings in each cohort.

Cohort			Cohort 1 + 2 n: 24 Patients					Cohort 1 n: 14 Patients					Cohort 3 n: 20 Patients					Overall Cohort: Cohort 1 + 2 + 3 n: 44 Patients				
Dose Map			Initial		Optimization		*p*	Initial		Optimization		*p*	Initial		Optimization		*p*	Initial		Optimization		*p*
			Mean	CI 95%	Mean	CI 95%		Mean	CI 95%	Mean	CI 95%		Mean	CI 95%	Mean	CI 95%		Mean	CI 95%	Mean	CI 95%	
PTV		PTV_95_ (%)	94.8	93.5-96.6	95.4	92.7–96.4	0.52	94.8	94.0–97.7	96.2	95.0–97.6	0.65	95.8	94.3–97.2	96.6	95.4–97.8	0.38	95.4	94.5–96.3	94.9	93.4–96.5	0.61
OAR	Pmap	DMean (Gy)	39.4	36.4–41.9	30.1	27.8–34.9	0.0001	36.6	32.9–40.5	27.7	26.7–29.8	<0.0001	14.6	11.1–18.1	10.3	6.8–13.8	0.08	28.8	24.2–33.4	22.1	18.3–26.0	0.03
	Spinal cord	DMax (Gy)	37.5	33.9–38.4	38.4	35.3–39.4	0.24	38.2	35.7–38.5	38.6	35.1–40.1	0.25	32.5	28.8–36.2	31.9	27.7–32.2	0.84	33.9	31.8–35.9	34.1	31.7–36.6	0.88
	LungH	DMean (Gy)	21.1	18.6–23.5	22.5	20.1–23.1	0.54	21.1	17.2–23.7	21.8	19.4–23	0.93	21.2	18.3–24.1	21.4	17.8–25.1	0.92	20.8	19.3–22.4	21.7	19.8–23.5	0.48
		DMax (Gy)	68	62.7–69.5	67.5	63.4–69.9	0.48	66.5	61.9–69.4	68.1	62.8–70.3	0.29	65.4	61.7–69.0	68.0	66.6–69.5	0.16	65.5	63.5–67.5	67.4	66.4–68.4	0.09
		V5% (%)	64.4	57.3– 72.0	71.3	63.9–80.4	0.22	64.9	57.6–80.9	69.1	59.1–92.4	0.58	68.2	61.6–74.7	63.6	53.4–73.7	0.43	67.1	62.5–71.7	68.2	62.3–74.0	0.77
		V10% (%)	56.2	49.3–61.6	59.8	52.2–66.8	0.27	56.2	50.3–68.8	58.5	52.2–82.1	0.68	59.9	53.6–66.2	56.0	46.5–65.5	0.48	58.1	53.9–62.3	58.6	53.5–63.8	0.88
		V13% (%)	51.5	40.1–57.5	52.7	49.1–60.9	0.32	52.2	48.1–62.6	51.3	48.5–67.2	0.78	54.5	48.1–60.8	51.9	43.0–60.7	0.62	52.2	48.3–56.1	53.4	48.8–58.0	0.69
		V20% (%)	39.2	34.3 – 46.0	40.5	36.9–45.7	0.39	39.2	33.3–46.1	37.5	36.4–47	0.68	41.6	35.3–47.8	41.4	34.2–48.6	0.97	40.1	36.8–43.5	41.8	38.1–45.6	0.50
		V30% (%)	28.1	25.8–32.4	28.8	24.8–34.4	0.65	26.4	20.5–33.8	27.6	23.9–35.4	0.55	28.3	23.0–33.6	29.8	24.0–35.6	0.68	28.3	25.4–31.3	30.1	27.1–33.2	0.40
	LungC	DMean (Gy)	7.7	5.8–10.3	8.2	6.3–10.5	0.85	7.2	5.4–11.2	9.1	6.3–13.2	0.52	8.7	7.4–10.0	7.7	6.4–9.1	0.26	8.4	7.5–9.4	8.2	7.1–9.3	0.74
		DMax (Gy)	48.2	34.0–56.6	49.8	43.1 – 62.0	0.71	47.6	22.0–55.3	51.2	40.9–62.6	0.58	51.7	44.0–59.5	53.6	46.3–61.0	0.71	47.9	42.4–53.4	50.4	45.2–55.5	0.51
		V5% (%)	55.4	43.7–62.7	58	46.4–64.7	0.93	57.3	36.9 – 76.0	61.1	46.5–91.8	0.82	57.3	48.8–65.8	49.5	40.0–59.1	0.21	56.4	50.8–62.1	53.4	46.6–60.2	0.49
		V10% (%)	32.4	15.1–38.9	34.5	17.4–38.8	0.95	28.5	12.1 – 43.0	37.5	17.4–44.3	0.46	36.1	29.3–42.8	30.9	24.8–37.1	0.25	58.1	53.9–62.3	58.6	53.5–63.8	0.88
		V13% (%)	18.4	8.9–28.5	19.4	11.5–27.2	0.86	15.3	5.3–33.7	19.4	12.2–34.8	0.35	25.8	19.6–32.1	21.2	16.9–25.5	0.21	52.2	48.3–56.1	53.4	48.8–58.0	0.69
		V20% (%)	4.6	1.9–9.7	5.4	2.4–11.9	0.6	4.3	0.3–9.4	6.1	2.2–17.1	0.27	9.5	6.3–12.7	8.4	6.0–10.8	0.56	40.1	36.8–43.5	41.8	38.1–45.6	0.50
		V30% (%)	1	0.1–3.1	1.2	0.4–4.5	0.66	0.9	0.0–3.5	1.2	0.4–5.1	0.38	2.9	1.4–4.4	2.7	1.4–4.0	0.81	28.3	25.4–31.3	30.1	27.1–33.2	0.40
	2Lungs	DMean (Gy)	15.0	12.6–16.7	16.0	14.2–16.6	0.38	15.2	12.2–16.7	16	12.6–17.7	0.31	15.6	13.1–18.0	13.6	11.7–15.5	0.20	14.9	13.7–16.2	14.6	13.5–15.8	0.72
		V13% (%)	35	29.2–42.9	38.2	33.7–43.1	0.29	33.5	28.3–44.1	40.1	30.5–45.5	0.41	38.6	34.5–42.8	34.3	29.1–39.5	0.19	36.4	33.6–39.2	36.8	33.2–40.3	0.88
		V20% (%)	22.9	21.3–26.3	26.6	21.7–28.5	0.22	22.2	20.9–26	24.7	20.9–32.3	0.33	24.7	21.8–27.6	23.1	19.9–26.4	0.45	24.1	22.4–25.9	24.8	22.7–26.8	0.63
		V30 (%)	15	13.2–19.4	18.4	14.9–19.5	0.43	13.7	11.2–19.5	18.2	12.5–21.1	0.38	14.8	12.5–17.1	14.8	12.4–17.2	1.00	15.5	14.2–16.9	16.2	14.8–17.7	0.47
	Heart	DMean (Gy)	9.8	5.5–12.3	10.9	7.6–16.4	0.4	11.3	6.2–18.9	12.1	6.8–18.3	0.78	10.1	7.3–12.9	8.6	5.3–11.9	0.47	9.9	8.1–11.8	10.2	8.1–12.2	0.87
		V30% (%)	5.6	1.7 – 13.0	9.4	4.3–11.5	0.45	5	1.7–14.2	8	4–11.9	0.52	9.3	5.9–12.7	8.6	4.8–12.4	0.75	9.6	6.3–12.8	9.5	7.0–12.0	1.00
		V40% (%)	3.5	0.7–4.9	4.2	1.9–7.1	0.26	2.8	0.7–4.9	3.6	1.7–7.2	0.55	5.1	3.3–6.8	5.1	2.9–7.2	1.0	4.5	3.2–5.8	5.5	3.9–7.1	0.32

Abbreviations: CI: Confidence Interval, OAT: Organ at Risk, PTV: Planning Target Volume, PTV_95:_ percentage of the PTV receiving 95% of the prescription dose, Pmap: Pmap region, DMean: mean dose received by the Volume of Interest (VOI), DMax: maximum dose received by the VOI, LungH: homolateral lung, LungC: contralateral lung, 2Lungs: both lungs, V_x_: percentage of the volume receiving x Gy.

**Table 2:** Results of the prediction model based on the initial and optimized plannings in each cohort.

Results of the Prediction Model		Cohort 1 + 2 n: 24 Patients			Cohort 1 n: 14 Patients			Cohort 3 n: 20 Patients			Cohort 1 + 2 + 3 n: 44 Patients		
		Initial Planning	Optimized Planning	*p*	Initial Planning	Optimized Planning	*p*	Initial Planning	Optimized Planning	*p*	Initial Planning	Optimized Planning	*p*
Risk probability of APT ≥ grade 2	Mean (%, SD)	90.8 (17.5)	60.9 (42.9)	0.003	93.9 (2.7)	43.9 (44.6)	0.0003	84.2 (26.0)	35.4 (17.6)	0.0001	87.8 (23.8)	45.3 (19.1)	<0.0001
	Median (%, CI 95%)	94.5 (92.9–95.4)	91.4 (7.9–93.7)	0.002	94.2 (91.9–95.5)	9.9 (7.5–93.6)	0.004	93.0 (89.8–94.1)	7.4 (5.2–89.1)	0.0001	93.9 (92.6–94.7)	9.1 (7.3–90.6)	<0.0001
Patients classified at high risk of APT (%)		100.0%	58.3%	0.001	100%	50%	0.006	100.0%	45.0%	0.0004	100%	56.8%	<0.0001
Mean IC		96.0	94.0	0.25	92.2	96.4	0.83	98.3	98.6	0.89	97.0	96.1	0.89
Mean HIV		94.0	94.0	0.77	94.3	95.5	0.36	93.5	94.2	0.59	93.8	93.9	0.87

Abbreviations: APT: Acute Pulmonary Toxicity, SD: Standard Deviation, CI: Confidence Interval, IC: Conformity Index, HIV: heterogeneity index.

- *A successful case (Patient #1):*
o *Patient’s characteristics: male patient, 74 years old; COPD: no; MEVS: 78.0% of the theorical value; history of smoking; chemotherapy: neoadjuvant and concurrent; AJCC stage: IIIA.*
o *3a (initial dose map): PTV_95_ = 96.4%; DMean_Pmap_ = 36.5 Gy; Prob_APT_ = 91.3%.*
o *3b (optimized dose map): PTV_95_ = 97.7%; DMean_Pmap_ = 24.8 Gy; Prob_APT_ = 7.5%.*
- *An unsuccessful case (Patient #6):*
o *Patient’s characteristics: male patient, 54 years old; COPD: no; MEVS: 46.8% of the theorical value; history of smoking; chemotherapy: neoadjuvant and concurrent; AJCC stage: IIIB.*
o *3c (initial dose map): PTV_95_ = 93.6%; DMean_Pmap_ = 60.2 Gy; Prob_APT_ = 97.7%.*
o *3d (optimized dose map): PTV_95_ = 68.3%; DMean_Pmap_ = 44.2 Gy; Prob_APT_ = 96.3%.*

## 4. Discussion

After an external validation of a prediction model of APT, we aimed to optimize a treatment plan in order to reduce the Dmean_Pmap_ and subsequently lower the patients’ risk of APT. From the initial cohort of 207 patients, 24 patients had a Dmean_Pmap_ above the predefined threshold and effectively presented an APT ≥ grade 2. Starting from the initial planning, the optimization was successful for 58.3% of the patients, meaning that the Dmean_Pmap_ significantly decreased under 30.3Gy and that the PTV_95_ coverage was maintained. For a subset of these patients (50.0%), the decrease in the Dmean_Pmap_ was associated with a change in the APT risk classification, with these patients being no longer classified at high risk of APT. For the 10 remaining patients (41.7%), no compromise between the Dmean_Pmap_ and the PTV_95_ was found. Based on the association of the DICE_PTV-Pmap_ and the success of the optimization (AUC 0.83), these patients appear to have a larger intersection between the PTV and the Pmap region. Interestingly, one patient from Cohort 2 was reclassified at low risk of APT thanks to the decrease of the Dmean_Pmap_. Despite a threshold having been previously set at 30.3Gy, the Dmean_Pmap_ appears to be predictive of APT even in patients with a lower Dmean_Pmap_.

In Cohort 3 (20 patients), optimizing the dosimetric planning resulted in a significant reduction of the APT risk, with 55.0% of these patients being reclassified as low risk patients. With a negative predictive value of 96.3% on the testing set [17], these results come with a certain robustness.

Other APT prediction tools were developed, such as radiomics-based tools based on the dose maps. A radiomics-based approach, while very efficient (a Bacc of 0.92 for the risk of APT ≥ grade 2) [23], remains limited to post-dosimetry evaluation. If a patient is categorized at high risk of APT based on the radiomics model, dosimetry planning must be renewed. With the voxel-based sub-region and the prediction model, constraint based on the Dmean can be defined a priori, with a risk deducted by combining the Dmean_Pmap_ and other DVH parameters. It appears as easily implementable in all centers treating lung cancer patients with VMAT.

Previous efforts were made regarding dosimetry planning with an adaptation to the functional sub-regions of the lungs. Higher doses to the perfused functional lung were a stronger predictor of toxicity than the dose to the conventionally measured lung [24]. Based on a cohort of 14 patients treated with 3D conformational radiotherapy, Siva et al. demonstrated proof of the principle that 3D conformational radiotherapy enables functional lung avoidance^12^. Similarly, the same team, using a ^68^Ga-ventilation/perfusion PET/CT, was able to optimize treatment planning with an avoidance of the perfused (Q) but not ventilated (V) lung sub-regions [15]. V/Q PET/CT functional volumes were strongly associated with pulmonary function tests [25]. While based on the physiological anatomy of the lungs, this approach requires performing high-resolution PET/CTs and has several limitations, among which are multi-modality registration and a smaller resolution. Furthermore, the clinical benefit of this optimization for a VMAT-based treatment remains yet to be proven [16]. Our approach relies on the retrospective analysis of a model developed on a large cohort of 165 patients and further prospectively validated on a cohort of 42 patients. In the subset of 24 eligible patients for this study, 33.3% of the patients were reclassified as low risks, thanks to the optimization of dosimetry planning.

Apart from the retrospective study and the relatively small cohort, the main limitation of our work is the inability to optimize the treatment for all included patients. For 41.7% of the patients in Cohorts 1 and 2, the optimization was unsuccessful at respecting both the dose constraints to the OARs and the PTV coverage. The APT risk classification only changed for a sub-set of these patients (33.3%), while a substantial number of patients from Cohort 3 benefited from a lower Dmean_Pmap_. Our approach appears to be both easily implementable on a daily basis and efficient at reducing the risk of APT without performing supplementary imaging. Regional radiosensitivity should be considered in usual lung dose constraints, opening the possibility of an easily implementable adaptive dosimetry plan. Lastly, the findings reported here might be important for an APT risk assessment also and especially in LALC cases amenable to limited re-irradiation to eradicate any residual cancer [26].

## 5. Conclusions

The proposed approach appears to be both easily implementable on a daily basis and efficient at reducing the predicted risk of APT. Regional radiosensitivity should be considered in usual lung dose constraints, opening the possibility of new treatment strategies, such as dose escalation or innovative treatment associations.

## Data Availability

Available on demand only.

## Supplementary Materials

The following supporting information can be downloaded at: https://www.mdpi.com/article/10.3390/cancers14153702/s1,; Supplementary Table S1: Dose constraints for the initial and optimized plannings; Supplementary Table S2: Individualized results of the prediction model based on the initial and optimized plannings; Supplementary Figure S1: Methodology flowchart for the fusion, dose map conversion and volume transfer; Supplementary Figure S2: Correlation between the DICEPTV-Pmap and the success of the dosimetric optimization—ROC curve; Supplementary Figure S3: Flowchart of the results regarding the APT risk classification.
