# Supplementary material for: VMAT-Based Planning Allows Sparing of a Spatial Dose Pattern Associated with Radiation Pneumonitis in Patients Treated with Radiotherapy for a Locally Advanced Lung Cancer
Source: Cancers (Basel). 2022 Jul 29;14(15):3702. doi: 10.3390/cancers14153702 (PMC9367460; doi:10.3390/cancers14153702)
Supplement: Supplementary file 1 [file cancers-14-03702-s001.zip › cancers-1805861-supplementary.pdf]

cancers

MDPI

Supplementary Materials

Supplementary Table S1. Dose constraints for the initial and optimized plannings.

Volume of Interest		Dose Constraint	Target	
			Initial Planning	OPTIMIZED Planning
Target volume	PTV	PTV_{95} (%)	≥ 95	≥ 95
OAR	LungH	V_{20} (%)	≤ 30	≤ 30
		V_{30} (%)	≤ 20	≤ 20
	LungC	V_{20} (%)	≤ 30	≤ 30
		V_{30} (%)	≤ 20	≤ 20
	2Lungs	V20 (%)	≤ 20%	≤ 20%
	Spinal cord	DMax (Gy)	≤ 45	≤ 45
	Heart	DMean (Gy)	≤ 5	≤ 5
		V_{30} (%)	≤ 100	≤ 100
		V_{40} (%)	≤ 30	≤ 30
	Pmap	DMean	-	< 30.3

Abbreviations: PTV: Planning Target Volume, PTV_{95}: volume of the PTV receiving 95% of the prescription dose, V_x: Percentage of the Volume receiving x Gy, DMax: maximum dose received by the volume of interest (VOI), DMean: mean dose received by the VOI.

Supplementary Table S2. Individualized results of the prediction model based on the initial and optimized plannings.

Patients's ID	Cohort	Initial Planning				Optimized Planning			
		$DMean_{Pmap}$	PTV_{95}	$Prob_{APT}$	$Pred_{APT}$	$DMean_{Pmap}$	PTV_{95}	$Prob_{APT}$	$Pred_{APT}$
Patient #1	1	36.5	96.4	91.3	APT	24.8	97.7	7.5	No APT
Patient #2	1	49.4	91.5	94.7	APT	30.3	90.7	7.9	No APT
Patient #3	2	36.2	98.6	9.2	APT	32.1	96.8	7.1	No APT
Patient #4	1	32.9	98.3	93.6	APT	29.8	96.2	93.6	APT
Patient #5	1	40.1	93.5	91.7	APT	30.0	91.1	11.8	APT
Patient #6	2	60.2	93.6	97.7	APT	44.2	68.3	96.3	APT
Patient #7	1	36.8	99.6	98.5	APT	27.6	97.6	97.0	APT
Patient #8	2	54.2	97.7	95.4	APT	37.5	89.6	92.9	APT
Patient #9	1	51.0	95.0	95.5	APT	26.8	98.4	3.1	No APT
Patient #10	2	39.3	93.1	93.9	APT	32.7	92.8	92.4	APT
Patient #11	1	38.8	94.7	95.7	APT	27.3	95.1	91.3	APT
Patient #12	1	44.0	94.0	94.9	APT	28.6	93.8	1.0	No APT
Patient #13	2	52.9	91.3	97.1	APT	41.0	88.3	94.4	APT
Patient #14	2	39.7	92.4	94.4	APT	39.7	92.4	90.6	APT
Patient #15	1	33.1	96.3	88.5	APT	30.3	96.4	7.2	No APT
Patient #16	2	40.5	96.3	94.6	APT	39.9	95.2	94.4	APT
Patient #17	2	41.5	96.4	97.3	APT	36.7	95.1	95.1	APT
Patient #18	1	30.6	98.3	95.1	APT	29.8	96.2	94.7	APT
Patient #19	2	36.7	97.0	92.7	APT	36.7	97.0	92.6	APT
Patient #20	2	43.0	91.6	93.3	APT	34.3	91.0	91.4	APT
Patient #21	1	32.1	97.7	92.1	APT	27.8	97.9	7.7	No APT
Patient #22	1	39.4	94.2	97.5	APT	25.5	95.6	93.9	APT
Patient #23	1	32.8	90.0	93.0	APT	27.5	95.7	89.6	APT
Patient #24	1	33.0	94.7	91.9	APT	26.0	96.7	7.9	No APT
Patient #25	3	13.2	84.4	91.3	APT	8.1	87.3	9.4	APT

Patient #26	3	6.7	99.3	95.2	APT	1.7	99.3	92.5	APT
Patient #27	3	26.2	94.2	94.1	APT	25.5	95.2	93.5	APT
Patient #28	3	6.1	94.3	89.5	APT	2.7	95.1	1.8	No APT
Patient #29	3	0.1	94.8	93.9	APT	0.1	95.7	2.3	No APT
Patient #30	3	16.5	94.6	95.9	APT	5.9	97.8	91.0	APT
Patient #31	3	14.5	96.0	92.1	APT	8.1	96.6	4.1	No APT
Patient #32	3	9.6	96.9	87.8	APT	6.9	97.3	5.2	No APT
Patient #33	3	13.9	94.6	95.0	APT	11.2	94.5	5.3	No APT
Patient #34	3	6.1	97.2	91.7	APT	4.5	95.7	90.5	APT
Patient #35	3	10.2	98.5	86.2	APT	6.4	97.6	3.6	No APT
Patient #36	3	17.1	94.8	87.3	APT	14.4	96.4	8.6	APT
Patient #37	3	17.4	96.5	95.6	APT	9.3	96.7	6.0	No APT
Patient #38	3	24.3	98.1	93.9	APT	19.9	98.7	87.7	APT
Patient #39	3	9.6	99.5	92.6	APT	5.6	99.6	6.9	No APT
Patient #40	3	21.3	97.3	96.7	APT	13.4	97.6	2.5	No APT
Patient #41	3	13.7	94.5	83.3	APT	7.5	96.4	89.4	APT
Patient #42	3	26.6	96.2	8.3	APT	26.4	96.3	7.5	No APT
Patient #43	3	12.9	95.7	94.0	APT	7.2	96.3	92.7	APT
Patient #44	3	26.1	97.8	8.8	APT	20.9	97.8	7.3	No APT

Abbreviations: $DMean_{Pmap}$: mean dose received by the Pmap-region, PTV_{95}: volume of the PTV receiving 95% of the prescription dose, $Prob_{APT}$: probability of APT, $Pred_{APT}$: classification prediction by the Pmap-model.

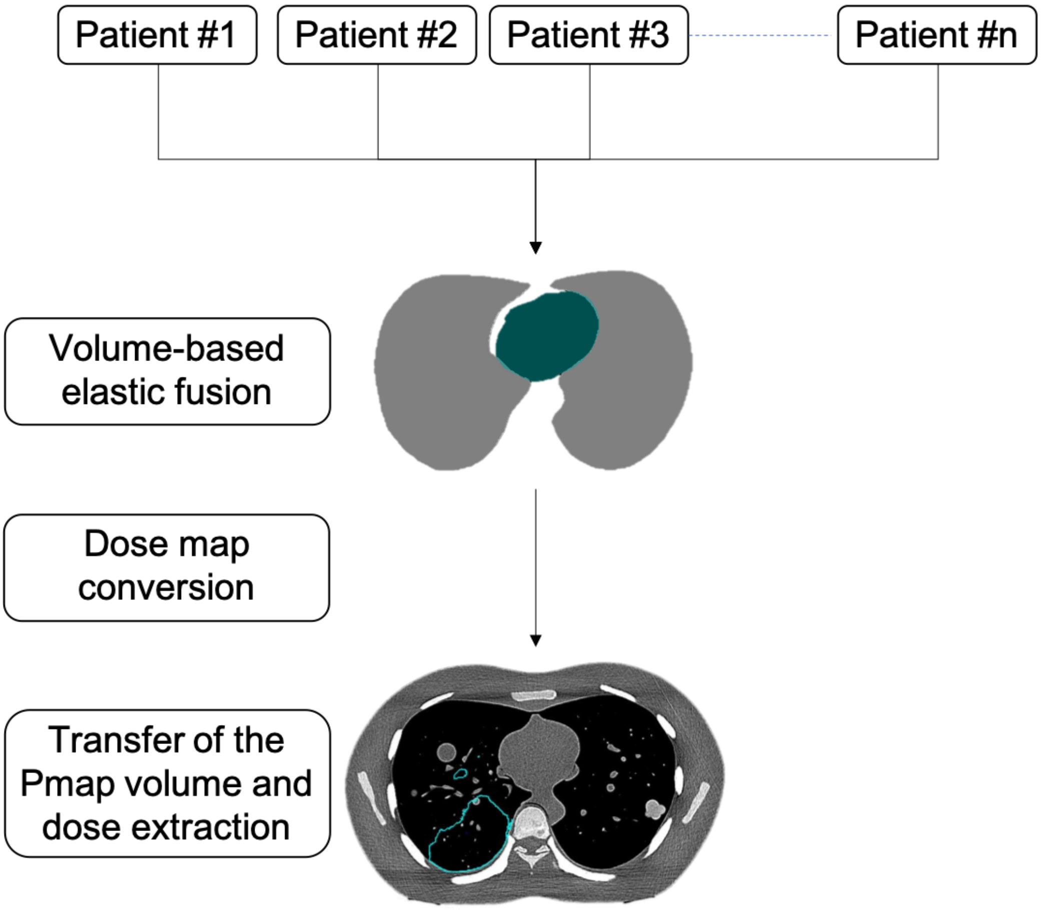

Supplementary Figure S1. Methodology flowchart for the fusion, dose map conversion and volume transfer.

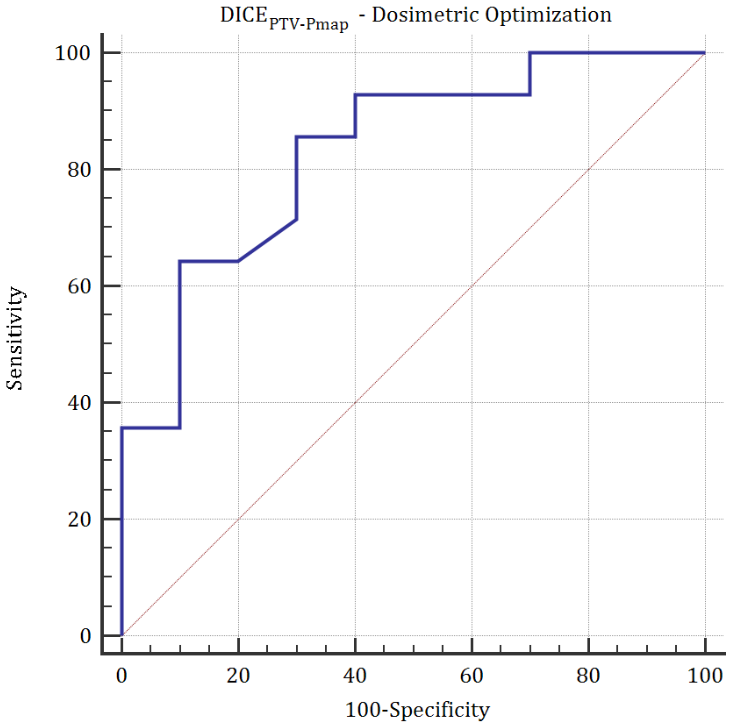

Supplementary Figure S2. Correlation between the DICE$_{PTV-Pmap}$ and the success of the dosimetric optimization – ROC curve.

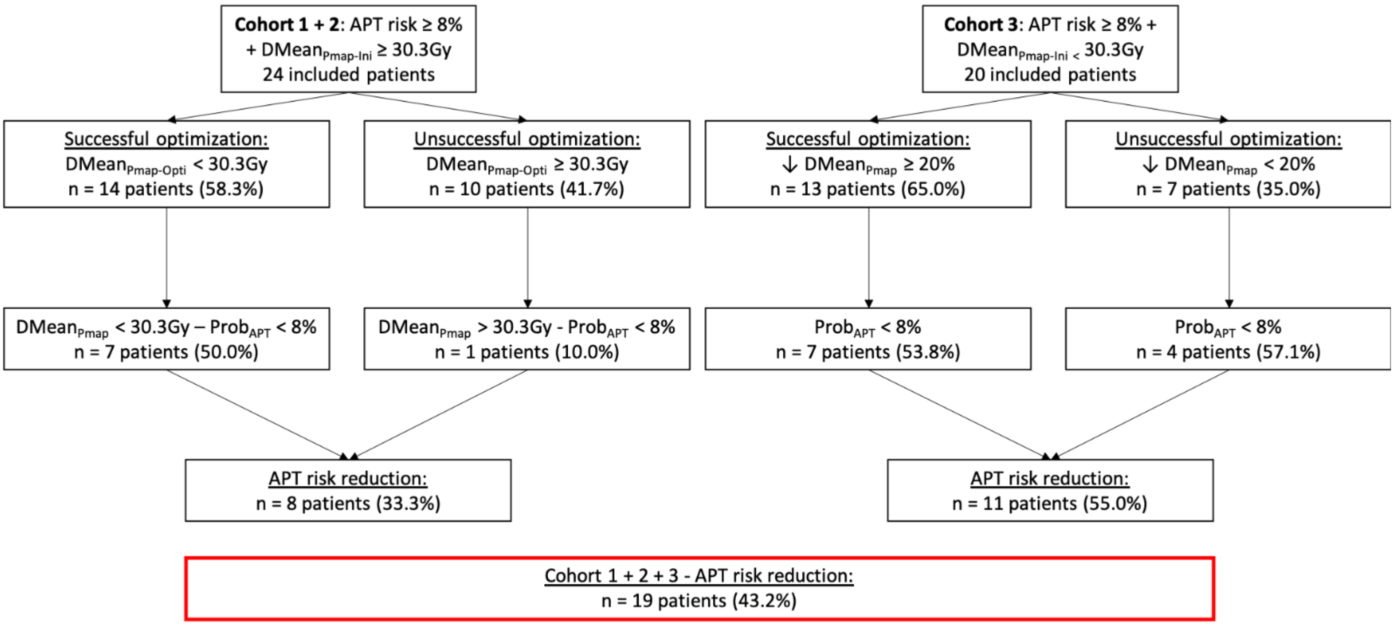

Supplementary Figure S3. Flowchart of the results regarding the APT risk classification. Abbreviations: APT: Acute Pulmonary Toxicity, DMean$_{Pmap}$: Mean Dose received by the Pmap-region, Prob$_{APT}$: probability of an APT ≥ grade 2 based on the Pmap-prediction model, DMean$_{Pmap-Ini}$: initial mean Dose to the Pmap region, DMean$_{Pmap-Opti}$: optimized mean Dose to the Pmap region.
